# Supplementary material for: HLA-B*27 Heavy Chain Homo-Oligomers Promote the Cytotoxicity of NK Cells via Activation of PI3K/AKT Signaling
Source: Medicina (Kaunas). 2022 Oct 7;58(10):1411. doi: 10.3390/medicina58101411 (PMC9608704; doi:10.3390/medicina58101411)
Supplement: Supplementary file 1 [file medicina-58-01411-s001.zip › medicina-1882661-supplementary.pdf]

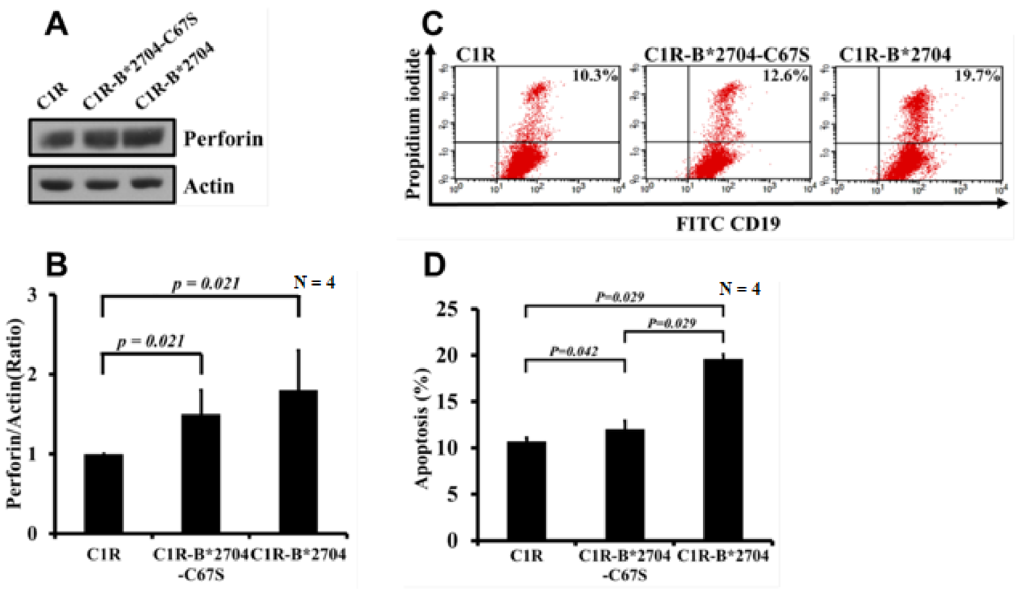

Supplementary Figure S1. The effect of co-culture with C1R, C1R-B*2704 C67S or C1R-B*2704 cells on the NK cell-mediated cytotoxicity. NK-92MI cells (1 x 10^6 cells) were co-cultured with C1R-B*2704 cells (2 x 10^5 cells), C1R-B*2704 C67S (2 x 10^5 cells) or C1R cells (2 x 10^5 cells) in MEM-α medium supplemented with sodium bicarbonate (1.5 g/L), 0.2 mM inositol, 0.02 mM folic acid, 0.01 mM 2-mercaptoethanol, 12.5% FBS , 12.5% horse serum and 5% CO_2 at 37℃. For western blotting and cytotoxicity assays, both cells co-cultured in the medium were carried for one day and 4 hours, respectively. (A) Western blotting analysis of the perforin expression in NK-92MI cells co-cultured with C1R, C1R-B*2704 C67S or C1R-B*2704. An aliquot (50 μg) of extracted proteins was separated by SDS-PAGE and analyzed by western blotting, probed for actin and perforin. (B) The ratio of perforin/actin averaged from four independent experiments in Figure 1A is plotted. (C) Analysis of NK-92MI-mediated cytotoxicity by flow cytometry. The apoptotic cells and C1R cells were stained by propidium iodide and anti-CD19 antibody, respectively. (D) The percentages of apoptotic cells induced by NK-92MI-mediated cytotoxicity averaged from four independent experiments in Fig. 1C are plotted.
